# Association of depression and sleep quality with frailty: a cross-sectional study in China

**DOI:** 10.3389/fpubh.2024.1361745

**Published:** 2024-04-05

**Authors:** Yue Zhang, Ge Yu, Wei Bai, Songyu Wu, Xiaohan Geng, Wangyi Zhang, Yihang Liu, Yujiao Meng, Junling Gao, Wenjun Li, Changgui Kou

**Affiliations:** ^1^Department of Epidemiology and Biostatistics, School of Public Health, Jilin University, Changchun, Jilin, China; ^2^School of Public Health, Fudan University, Shanghai, China; ^3^Department of Social Medicine and Health Management, School of Public Health, Jilin University, Changchun, Jilin, China

**Keywords:** frailty, depression, sleep quality, older adults, community, mediation effect

## Abstract

**Background:**

With the rapid growth of global aging, frailty has become a serious public health burden, affecting the life quality of older adults. Depressive symptoms (depression hereafter) and sleep quality are associated with frailty, but the pathways in which sleep quality and depression affect frailty remain unclear.

**Method:**

This cross-sectional study included 1866 community-dwelling older adults. Demographic characteristics and health-related data of them was collected, and we also assessed frailty, depression, and sleep quality. Descriptive statistics were carried out and ordinal logistic regression analysis was used to identify the factors correlated with frailty. Spearman correlation analysis and mediation analysis were employed to assess associations between sleep quality, depression and frailty. Two-sided *p* < 0.05 was considered as significant.

**Results:**

The results showed that 4.1% older adults were frail and 31.0% were pre-frail. Ordinal logistic regression showed that age, consumptions of vegetables, exercise, sleep quality, depression, number of chronic diseases, chronic pain, and self-rated health were correlated with frailty. Spearman correlation analysis revealed that frailty was associated with depression and sleep quality. There was a mediation effect that sleep quality was a significant and positive predictor of frailty (total effect = 0.0545, 95% boot CI = 0.0449–0.0641), and depression was a mediator between sleep quality and frailty (mediation effect = 60.4%).

**Conclusion:**

Depression and poor sleep quality may be early indicators of frailty in older adults. Improving the sleep quality and psychological state of older adults can improve frailty, which is beneficial for healthy aging.

## 1 Introduction

Population aging is an increasing global social concern (1). The proportion of the population over 65 years old is expected to rise from 10% in 2022 to 16% by 2050 (2). Physical function varies among older adults, with some older adults being dependent and limited by disease or disability (3). Severe functional impairments can lower life quality in older adults, and occupy medical financed or community care systems (4, 5). Therefore, while prolonging remaining years of life of older adults, more attention should be paid to extending healthy life expectancy (6).

Frailty of older adults is a new frontier in medicine, often seen as a precursor to age-related diseases (7, 8). It describes a clinical state of decompensation in the presence of stressors, as a result of the unstable homeostasis of body composition and physiological systems due to multisystem changes (9). This implies that minor stressor events could also lead to major changes in the health status of the older adults with frailty (10). Frailty is considered as a biological syndrome, and includes five physical components, which are fatigue, resistance, ambulation, illness, and loss of weight. Frailty is considered when three or more of these components are present. Pre-frailty is the classification given when one or two of the components, and robust is given to an individual who has no frailty component (10, 11). A previous study in Chinese older adults found that 27.5% were frail, and 51.3% were pre-frail (12). Besides, frailty is a dynamic process, which means that pre-frail older adults may progress to a robust or frail state, but more commonly, the transition tends to be in a worse direction (10, 13). A 10-year prospective cohort study proved that frailty was the principal cause of death (14). Additionally, a meta-analysis indicated that reducing frailty could effectively improve life quality in older adults (15). Hence, it is vital to identify the high-risk population with frailty and provide early intervention to them.

According to current research, psychological problems and sleep problems in older adults were related to frailty (16, 17). Depression is widely prevalent among older adults, and its presentation and outcomes differ from those of young people. A study reported that 40.4% of older adults with depression also had frailty, and the odds of experiencing frailty increased compared with those who did not have depression (18). Sleep disturbances and fatigue are widespread in older adults (19). As people grow older, sleep patterns tend to change gradually, which may affect their ability to fall asleep and stay asleep (20). A prospective study discovered that keeping healthy sleep patterns contributed to a lower risk of being frail and pre-frail, and sleep difficulties were associated with frailty (21). However, some studies demonstrated a potential bidirectional association between depression and sleep quality, in which sleep disturbances might exacerbate depression, and older adults with depression often experienced poor sleep quality (22–24). Furthermore, depression and sleep disturbances usually exist as comorbidities (25). Sleep disturbance is a core symptom of depression, and more than 90% of patients with depression have sleep disturbances (26).

Given the aforementioned, frailty had a major impact on older adults, but unlike certain irreversible physical disabilities, it could be prevented and reversed. Psychological and sleep problems were closely associated with frailty. Addressing the psychological and sleep issues of older adults provides a solution to dealing with frailty. However, few studies have investigated the paths in which sleep and depression affect frailty (27). Hence, the objectives of this study were to explore the mediating effect of depression and sleep quality on frailty, and analyzing the factors that increase the risk of frailty in older adults.

## 2 Materials and methods

### 2.1 Data

Data were collected from 10 communities in Changchun, Jilin Province, China between July 2022 and September 2022. The participants were included if they were older adults aged 65 or older, were willing to participate, lived in the community for at least 6 months, had clear consciousness, and communicated normally. We excluded those with severe cognitive dysfunction or physical illness, and questionnaires that did not pass consistent quality control measures. All participants or their respondents provided informed consent. The study finally included 1866 participants after excluding 152 invalid questionnaires. Considering the simultaneity of the implementation of the study with the COVID-19 pandemic, both investigators and participants were with necessary precaution to avoid the risk of contagion. Moreover, at the time of the investigation, the region was not experiencing a pandemic outbreak, and the permanent residents had largely returned to their normal routines. Therefore, face-to-face interviews could be carried out normally. During face-to-face interviews with, investigators who received standardized training completed the questionnaires based on the answers provided by the older adults at community health service centers (CHSC). The gathered data was entered into digital survey forms. Before conducting formal investigations, preliminary investigations were carried out to ensure the feasibility and appropriateness of the projects. Participants completed the informed consent, and were informed that there were no unknown risks present throughout the study to promote cooperation. The filling process of the questionnaires was conducted objectively.

### 2.2 Basic characteristics

Sociodemographic and health-related data were obtained using a structured questionnaire: (i) age, gender, nation, education, marital status, live alone, and monthly income; (ii) smoking, drinking, vegetables, fruits, and exercise; (iii) number of chronic diseases, chronic pain, and self-rated health. Smoking and drinking were determined by cigarette consumption and drinking frequency, respectively. Based on the Dietary Guidelines for Chinese Residents (28) and recommendations of the EAT-Lancet Commission (29), consumptions of vegetables were divided into the following groups based on weight of uncooked edible portions per day: 0–200 g, 201–300 g, 301–500 g, and more than 500 g. Similarly, consumptions of fruits were categorized into the following groups by edible portion: 0–100 g, 101–200 g, 201–350 g, and more than 350 g (28, 29). Exercise was defined as physical activities, such as dancing or exercises, which could cause a faster heartbeat or mild sweating. Chronic diseases referred to diseases previously diagnosed by hospitals. Chronic pain was considered as self-perceived chronic pain with a frequency of at least 3–4 times a week, and self-rated health was classified as good, moderate, or bad.

### 2.3 Questionnaires

Frailty was assessed using the Fatigue, Resistance, Ambulation, Illness and Loss of Weight Index (FRAIL), which was developed in 2008 by the International Association of Nutrition and Aging (30), and modified by Morley et al. (11). Compared with other short screening tools, FRAIL had good performance in frailty (31). The scale contained 5 items: (i) fatigue: feeling tired most of the time in the past month; (ii) resistance: feeling difficult to climb 10 steps by yourself and not using aids; (iii) ambulation: unable to walk continuously for 100 meters independently; (iv) illness: more than 5 illnesses; (v) loss of weight: weight loss of unknown reason more than 5% within 1 year. Each item was answered using a dichotomous “yes” or “no” response, and 1 point was given if the answer was “yes.” The total score ranged from 0 to 5, with 0 as not-frail, 1–2 points as pre-frail, and ≥3 points as frail. The Cronbach’s alpha was 0.716 in this study.

The Pittsburgh Sleep Quality Index (PSQI) was used to measure sleep quality which was designed by Buysse et al. (32). The scale included a total of 24 items, with 19 self-rated questions used for scoring. The PSQI was divided into 7 component scores, including sleep latency, sleep duration, sleep disturbances, subjective sleep quality, use of sleeping medications, habitual sleep efficiency, and daytime dysfunction. The score of each component was 0–3 points, and the total score of PSQI was the sum of the above 7 component scores, resulting in a range of 0–21 points. Participants were considered as poor sleep quality when the total score of PSQI was above 5. A higher score indicates worse sleep quality. The Cronbach’s alpha was 0.715 in this study.

The Patient Health Questionnaire-9 (PHQ-9), a concise and valid tool to identify the severity of depressive symptoms, was developed by Columbia University in the United States, and has been widely used for depression screening (33, 34). The scale consisted of 9 items, with each item scoring from 0 (not at all) to 3 (nearly every day). The total scores ranged from 0 to 27, which was divided into normal (0–4), mild depression (5–9), moderate depression (10–14), moderate severe depression (15–19), and severe depression (20–27). The PHQ-9 was found to have excellent internal reliability (35), with a Cronbach’s alpha of 0.882 in this study.

### 2.4 Statistical analysis

Sociodemographic characteristics and health-related factors used in this study were all categorical variables, and descriptive statistics were utilized to summarize. Frailty was classified it into three states: robust, pre-frail, and frail, with a hierarchical relationship among them. Distributions of frailty status were compared across different groups (i.e., demographic variables) using Mann–Whitney *U* test or Kruskal–Wallis test as appropriate. A multivariate analysis was performed by ordinal logistic regression to evaluate the risk factors for frailty in older adults. The test of parallel lines was used to assess whether the proportional odds assumption was violated in the ordinal logistic regression model. Spearman correlation analysis was used to find whether there was a connection between sleep quality, depression, and frailty. Moreover, mediation analysis was carried out, and 95% confidence intervals (CI) were assessed using bootstrapping (5,000 bootstrapped samples). SPSS (Version 24.0) and R (version 4.2.2), and PROCESS Procedure for SPSS v3.5 were used. A significant level was set at 2-sided *p* < 0.05.

## 3 Results

### 3.1 Demographic characteristics

The results showed that 53.1% participants were 65–69 years old. And the participants were classified into three frailty status, non-frail (64.9%), pre-frail (31.0%), and frail (4.1%). As is shown in Table 1, differences were significant in different age groups across three frailty status groups ( $\chi^{2}$ =9.480, *p* = 0.024). Female participants had higher ratios of pre-frailty and frailty compared to male participants (*Z* = −2.723, *p* = 0.006). Among participants with a college degree and above, the prevalence of pre-frailty was higher, with a prevalence of 40.2%, than in other participants. However, there was no significant effect of nation, marital status, live alone, and monthly income across three frailty status groups.

**Table 1:** Distributions of frailty status across different demographic characteristics.

Variable		Overall *n* (%)	Frailty status			*Z/*χ^2^	*p*
			Non-frail *n* (%)	Pre-frail *n* (%)	Frail *n* (%)		
Overall	–	1866 (100.0)	1,211 (64.9)	578 (31.0)	77 (4.1)	–	–
Age, years	65–69	990 (53.1)	656 (66.3)	295 (29.8)	39 (3.9)	9.480	0.024
	70–74	421 (22.6)	285 (67.7)	121 (28.7)	15 (3.6)		
	75–79	197 (10.6)	122 (61.9)	65 (33.0)	10 (5.1)		
	≥80	258 (13.8)	148 (57.4)	97 (37.6)	13 (5.0)		
Gender	Man	619 (33.2)	429 (69.3)	166 (26.8)	24 (3.9)	−2.723	0.006
	Woman	1,247 (66.8)	782 (62.7)	412 (33.0)	53 (4.3)		
Nation	Han	1779 (95.3)	1,150 (64.6)	555 (31.2)	74 (4.2)	−1.038	0.299
	Others	87 (4.7)	61 (70.1)	23 (26.4)	3 (3.4)		
Education	Primary school and below	377 (20.2)	239 (63.4)	118 (31.3)	20 (5.3)	14.098	0.003
	Junior middle school	758 (40.6)	511 (67.4)	222 (29.3)	25 (3.3)		
	Senior middle school/technical secondary school	487 (26.1)	327 (67.1)	140 (28.7)	20 (4.1)		
	College degree and above	244 (13.1)	134 (54.9)	98 (40.2)	12 (4.9)		
Marital status	Married	1,306 (70.0)	862 (66.0)	386 (29.6)	58 (4.4)	−1.298	0.194

Not married	560 (30.0)	349 (62.3)	192 (34.3)	19 (3.4)		
Live alone	Yes	356 (19.1)	235 (66.0)	109 (30.6)	12 (3.4)	−0.580	0.562

No	1,510 (80.9)	976 (64.6)	469 (31.1)	65 (4.3)		
Monthly income, yuan	<2000	425 (22.8)	271 (63.8)	137 (32.2)	17 (4.0)	4.017	0.134

2000–5,000	1,261 (67.6)	835 (66.2)	373 (29.6)	53 (4.2)		

>5,000	180 (9.6)	105 (58.3)	68 (37.8)	7 (3.9)		

### 3.2 Social and health factors

**Figure 1:**
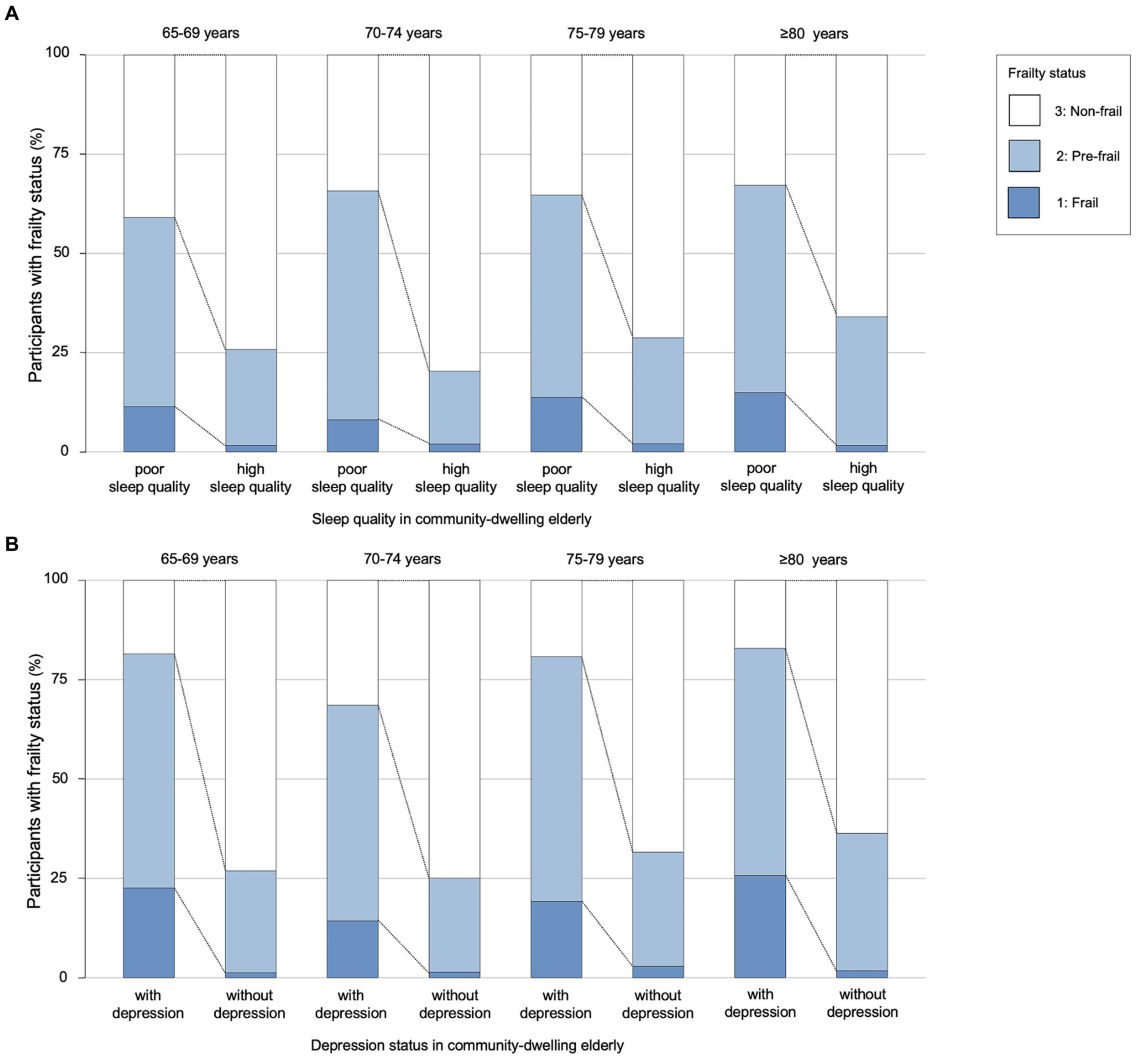
The association between sleep quality, depression and frailty in four age groups. **(A)** Sleep quality in community-dwelling older adults. **(B)** Depression status in community-dwelling older adults. The older adults were divided into four age groups. Each group was divided by sleep quality in **(A)** and by depression status in **(B)**. There were significant differences in the prevalence of frailty between the sleep quality groups and the depression status groups of older adults.

The results showed that there were significant differences between non-frail, pre-frailty, and frailty participants in terms of their daily consumption of vegetables ( $\chi^{2}$ = 27.395, *p* < 0.001) and fruits ( $\chi^{2}$ = 15.802, *p* = 0.001). Prevalence of pre-frailty (29.6%) and frailty (3.6%) was lower in participants who usually exercise, compared to those who did not (*Z* = −4.864, *p* < 0.001). In addition, results indicated that depression was also a significant factor across three frailty status groups (*Z* = −16.710, *p* < 0.001). Furthermore, participants with pre-frailty and frailty tended to have poorer sleep quality (*Z* = −14.868, *p* < 0.001). The results also revealed significant differences in the number of chronic diseases ( $\chi^{2}$ = 218.491, *p* < 0.001), chronic pain (*Z* = −17.724, *p* < 0.001), and self-rated health ( $\chi^{2}$ = 266.489, *p* < 0.001) across different status of frailty (Table 2 and Figure 1).

**Table 2:** Distributions of frailty status across different social and health factors.

Variable		Overall *n* (%)	Frailty status			*Z/*χ^2^	*p*
			Non-frail *n* (%)	Pre-frail *n* (%)	Frail *n* (%)		
Smoking	Yes	352 (18.9)	236 (67.0)	97 (27.6)	19 (5.4)	−0.697	0.486
	No	1,514 (81.1)	975 (64.4)	481 (31.8)	58 (3.8)		
Drinking	Yes	268 (14.4)	171 (63.8)	89 (33.2)	8 (3.0)	−0.237	0.812
	No	1,598 (85.6)	1,040 (65.1)	489 (30.6)	69 (4.3)		
Vegetables, gram	0–200	87 (4.7)	37 (42.5)	39 (44.8)	11 (12.6)	27.395	<0.001
	201–300	440 (23.6)	275 (62.5)	141 (32.0)	24 (5.5)		
	301–500	651 (34.9)	437 (67.1)	192 (29.5)	22 (3.4)		
	≥501	688 (36.9)	462 (67.2)	206 (29.9)	20 (2.9)		
Fruits, gram	0–100	411 (22.0)	236 (57.4)	151 (36.7)	24 (5.8)	15.802	0.001
	101–200	541 (29.0)	374 (69.1)	150 (27.7)	17 (3.1)		
	201–350	543 (29.1)	355 (65.4)	161 (29.7)	27 (5.0)		
	≥351	371 (19.9)	246 (66.3)	116 (31.3)	9 (2.4)		
Exercise	Yes	1,629 (87.3)	1,089 (66.9)	482 (29.6)	58 (3.6)	−4.864	<0.001
	No	237 (12.7)	122 (51.5)	96 (40.5)	19 (8.0)		
Sleep quality	High	1,400 (75.0)	1,036 (74.0)	340 (24.3)	24 (1.7)	−14.868	<0.001
	Poor	466 (25.0)	175 (37.6)	238 (51.1)	53 (11.4)		
Depression	Yes	255 (13.7)	56 (22.0)	147 (57.6)	52 (20.4)	−16.710	<0.001
	No	1,611 (86.3)	1,155 (71.7)	431 (26.8)	25 (1.6)		
Number of chronic diseases	0	576 (30.9)	473 (82.1)	100 (17.4)	3 (0.5)	218.491	<0.001

1	455 (24.4)	340 (74.7)	113 (24.8)	2 (0.4)		
	≥2	835 (44.7)	398 (47.7)	365 (43.7)	72 (8.6)		
Chronic pain	Yes	452 (24.2)	145 (32.1)	243 (53.8)	64 (14.2)	−17.724	<0.001
	No	1,414 (75.8)	1,066 (75.4)	335 (23.7)	13 (0.9)		
Self-rated health	Good	1,091 (58.5)	838 (76.8)	246 (22.5)	7 (0.6)	266.489	<0.001
	Moderate	645 (34.6)	350 (54.3)	261 (40.5)	34 (5.3)		
	Bad	130 (7.0)	23 (17.7)	71 (54.6)	36 (27.7)		

### 3.3 Ordinal logistic regression analysis

In the ordinal logistic regression analysis, age ≥ 80 displayed a significant increase in the ordinal frailty status (OR = 1.474, 95% CI = 1.065–2.040). Daily consumption of vegetables more than 300 g had positive effects on pre-frailty and frailty. Exercise was associated with decreased risks of pre-frailty and frailty (OR = 0.562, 95% CI = 0.414–0.762). Poor sleep quality (OR = 1.795, 95% CI = 1.373–2.346), and depression (OR = 3.899, 95% CI = 2.783–5.464) were found to be associated with increased risks. Significant associations between number of chronic diseases, chronic pain, self-rated health and frailty status were also found, indicating that number of chronic diseases (≥2) (OR = 2.248, 95% CI = 1.659–3.047), participants experiencing chronic pain (OR = 3.322, 95% CI = 2.557–4.315), and a moderate (OR = 1.397, 95% CI = 1.084–1.801) or bad health state (OR = 5.795, 95% CI = 3.734–8.995) had negative effects on frailty status. However, no statistically significant differences were found in gender, education, and fruits in the ordinal frailty status. The results of test of parallel lines showed that the model followed the proportional odds assumption (*p* = 0.200) (Table 3 and Supplementary Figure S1).

**Table 3:** Model of factors associated frailty.

Variable	*β*	*SE*	Waldχ^2^	*p*	OR (95%CI)
Age, years					
65–69	–	–	–	–	1.000
70–74	−0.366	0.147	6.228	0.013	0.694 (0.520–0.924)
75–79	0.076	0.183	0.172	0.678	1.079 (0.754–1.543)
≥80	0.388	0.166	5.462	0.019	1.474 (1.065–2.040)
Gender					
Man	–	–	–	–	1.000
Woman	−0.021	0.123	0.029	0.865	0.979 (0.770–1.246)
Education					
Primary school and below	–	–	–	–	1.000
Junior middle school	−0.074	0.156	0.228	0.633	0.928 (0.684–1.259)
Senior middle school/ technical secondary school	−0.203	0.171	1.411	0.235	0.816 (0.584–1.141)
College degree and above	0.044	0.194	0.052	0.819	1.045 (0.715–1.529)
Vegetables, gram					
0–200	–	–	–	–	1.000
201–300	−0.501	0.264	3.608	0.058	0.606 (0.361–1.016)
301–500	−0.581	0.261	4.962	0.026	0.559 (0.335–0.933)
≥501	−0.694	0.264	6.915	0.009	0.499 (0.298–0.838)
Fruits, gram					
0–100	–	–	–	–	1.000
101–200	−0.230	0.160	2.082	0.149	0.794 (0.581–1.086)
201–350	0.074	0.159	0.214	0.644	1.076 (0.788–1.471)
≥351	0.149	0.182	0.674	0.412	1.161 (0.813–1.657)
Exercise					
No	–	–	–	–	1.000
Yes	−0.576	0.156	13.718	*<*0.001	0.562 (0.414–0.762)
Sleep quality					
High	–	–	–	–	1.000
Poor	0.585	0.137	18.288	*<*0.001	1.795 (1.373–2.346)
Depression					
No	–	–	–	–	1.000
Yes	1.361	0.172	62.490	*<*0.001	3.899 (2.783–5.464)
Number of chronic diseases					
0	–	–	–	–	1.000
1	0.178	0.167	1.133	0.287	1.194 (0.861–1.657)
≥2	0.810	0.155	27.299	*<*0.001	2.248 (1.659–3.047)
Chronic pain					
No	–	–	–	–	1.000
Yes	1.201	0.134	80.919	*<*0.001	3.322 (2.557–4.315)
Self-rated health					
Good	–	–	–	–	1.000
Moderate	0.335	0.129	6.692	0.010	1.397 (1.084–1.801)
Bad	1.757	0.224	61.370	*<*0.001	5.795 (3.734–8.995)

OR, odds ratio; CI, confidence intervals.

### 3.4 Spearman correlation analysis and mediation effect

**Figure 2:**
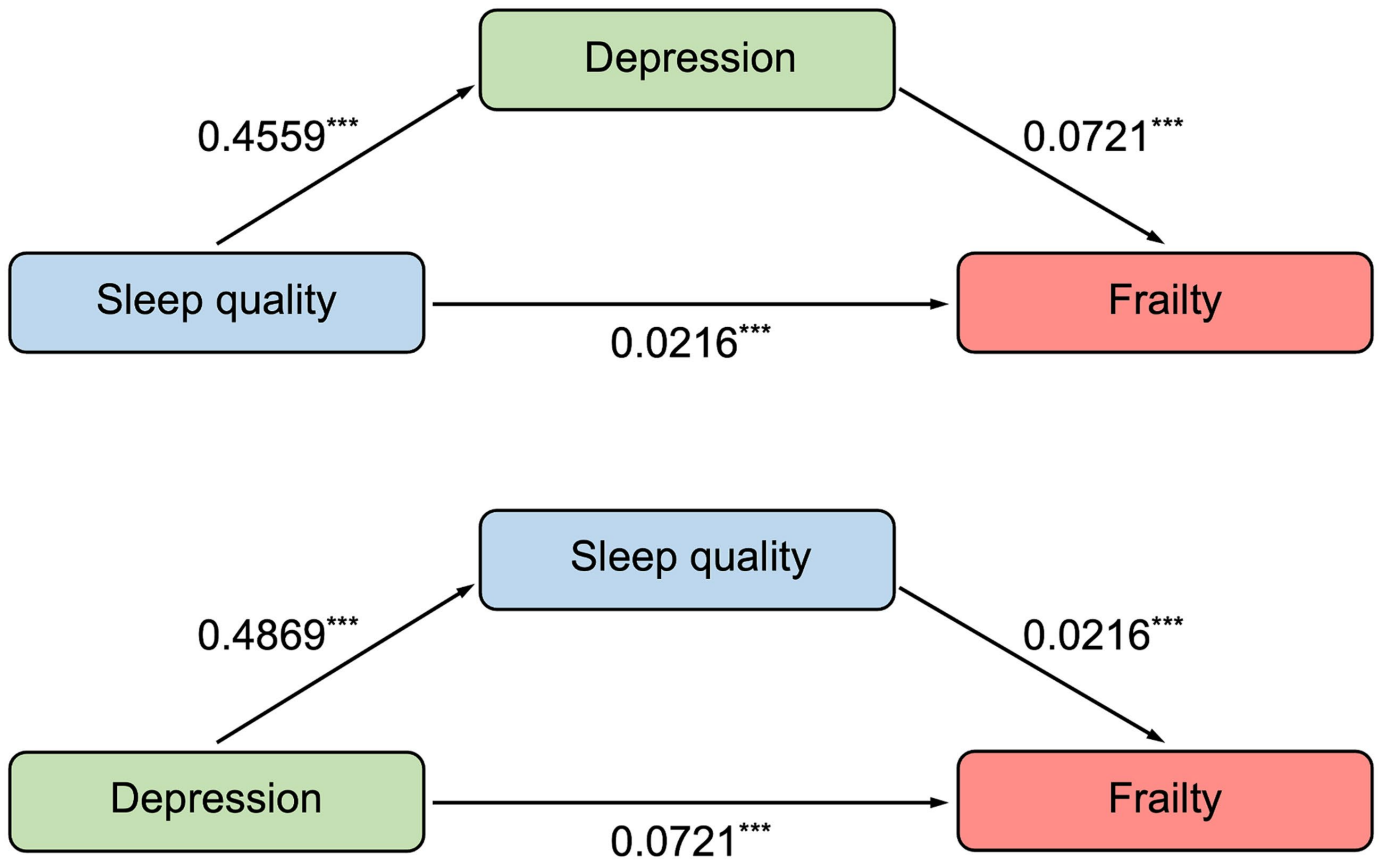
The mediation model of sleep quality and depression to frailty. ^***^p < 0.001. Results of mediation analysis showed that there was a mediation effect that sleep quality was a significant and positive predictor of frailty (direct effect = 0.0216), with depression being a mediator between sleep quality and frailty. And considering the bidirectional effect of depression and sleep quality, we also examined the mediation effect between depression and frailty (direct effect = 0.0721), with sleep quality as a mediator.

Spearman correlation analysis revealed that participants with depression had worse sleep quality (*r* = 0.602, *p* < 0.001) and higher frailty (*r* = 0.492, *p* < 0.001). In addition, there was a correlation between poor sleep quality and frailty (*r* = 0.403, *p* < 0.001), presented in Table 4. In this study, age, vegetables, exercise, number of chronic diseases, chronic pain, and self-rated health were associated with frailty through ordinal logistic regression, which we deemed as covariates. And according to previous research (36), we also included gender as a covariate. After controlling for covariates, results of mediation analysis revealed that poor sleep quality was a significant positive predictor of frailty (total effect = 0.0545, 95% boot CI = 0.0449–0.0641). The indirect effect of poor sleep quality on frailty mediated by depression was found (indirect effect = 0.0329, 95% boot CI = 0.0246–0.0416), and the indirect effect accounted for 60.4% of the total effect of poor sleep quality on frailty. Considering the bidirectional effect of depression and sleep quality, the study also selected sleep quality as a mediator variable. The direct effect of depression on frailty was 0.0721 (95% boot CI = 0.0614–0.0829), which accounted for 87.3% of the total effect of depression on frailty (total effect = 0.0826, 95% boot CI = 0.0731–0.0922) (Supplementary Table S1 and Figure 2).

**Table 4:** Spearman correlation coefficients between depression, sleep quality and frailty.

Variable	Depression	Sleep quality	Frailty
Depression	1		
Sleep quality	0.602^***^	1	
Frailty	0.492^***^	0.403^***^	1

^***^p < 0.001.

## 4 Discussion

The study found that depression mediated the effect of sleep quality on frailty, and sleep quality also mediated the effect of depression on frailty. The latter had a weaker effect than the former. The results indicated that roughly 35% of community-dwelling older adults were either frail or pre-frail. Demographic characteristics and health-related factors were associated with pre-frailty and frailty in older adults.

Results showed that 4.1% of participants in Chinese community-dwelling older adults were frail, and 31.0% were pre-frail, which was similar to Li’s study (37), but lower than the findings reported in other previous studies (38–40). Differences in the tools used for assessing frailty may explain this variation (31). The prevalence of frailty in older adults also differed across various regions and cultures (36, 41). Moreover, the analyzed participants may differ in age and other factors from previous studies. In this study, age, vegetables, exercise, sleep quality, depression, number of chronic diseases, chronic pain, and self-rated health had associations with frailty, which had also been supported by previous studies (42, 43). Although, some studies had suggested a connection between gender and frailty (44, 45), this study did not support this finding. In addition, after adjusting for covariates, education level was no longer significantly associated with frailty in older adults. The effect of education level as a social factor on frailty varies among individuals. Contrary to some previous studies, daily consumption of fruits did not have significant associations with frailty in this study (44, 46). Several studies have shown that poor nutrition has been identified as a risk factor for frailty (47, 48). However, nutrition may not be entirely equivalent to the consumption of fruits and vegetables, and older adults may consume dairy products and nutraceuticals in addition to meals to supplement their nutritional needs. Additionally, dietary intervention is usually combined with exercise training, and there is insufficient evidence to suggest that diet alone can prevent or cure frailty (48).

Consistent with previous studies, the prevalence of pre-frailty was higher than that of frailty (12, 37). Similar to frailty, pre-frailty is also associated with an increased risk of psychological distress, diabetes, multiple sclerosis, stroke, myocardial infarction, and other chronic diseases, and mortality (49, 50). According to the findings, 31.0% of older adults aged ≥65 in the community had pre-frailty. Given the dynamics of frailty, it is important to pay attention to the pre-frailty, as it has the potential to advance to a worse state of frailty at any given moment (51, 52). Some studies suggested that frailty should be seen as a continuous procession, with the occurrence of frailty being associated with an increased risk of adverse outcomes, and there was a dose–response association between frailty and adverse outcomes (51, 52). Lifestyle or behavioral interventions, a balanced diet, regular exercise, and increased social engagement can aid in the prevention of frailty (53, 54). It is, therefore, advisable for a rapid screening test for frailty to be included in routine community screening programs, which contributes to timely management of continuous and dynamic frailty process in older adults, and provides appropriate intervention measures. This work has the potential to alleviate the economic and psychological strain of frailty on individuals, families, and caregivers, as well as reduce the burden on the national healthcare system.

Older adults residing in communities with poorer sleep quality were at greater risk of frailty. The prevalence of frailty in older adults with poor sleep quality was 1.795 times higher than those with good sleep quality, consistent with prior research (17). Older adults with sleep disorders may experience adverse outcomes such as decreased grip strength, fatigue, and slow walking speed, which are typical symptoms of frailty (55–57). Studies have shown that a correlation between prolonged or decreased sleeping hours and frailty (17, 58). In addition to sleep duration, the components of sleep quality in PSQI, such as sleep latency, sleep disturbance, poor subjective sleep quality, sleep efficiency, and daytime dysfunction components have also been proven to be associated with frailty (59). Thus, it is important for older adults experiencing a decline in sleep quality to be alert to the possibility of developing or worsening frailty.

Depression in older adults is also associated with frailty. Given the results of this study, 13.7% of older adults experienced depression. Older adults with depression in the community were 3.8 times more likely to suffer from frailty than those without depression, which was consistent with a previous prospective cohort study (16). Studies have demonstrated that older adults with depression and frailty are more likely to develop suicidal ideation, which poses a serious threat to their safety (60). Common symptoms of depression include low mood, cognitive decline, worsening physical illness, decreased positive affect or pleasure in response to social contacts and routine activities, and social isolation or withdrawal (61). Such symptoms often lead to a decrease in social activity in older adults with depression and may trigger sarcopenia, thus increasing the likelihood of frailty in this population (62, 63). Furthermore, chronic pain was identified to be correlated with of frailty. Previous studies have shown that depression may play a mediating role, and that non-frail older adults with chronic pain are more likely to experience physical frailty after a follow-up period (64). Chronic pain in older adults should be effectively intervened, because it may alleviate their depression and improve their frailty. Additionally, exercise has been shown to effectively treat depression and is associated with frailty (65, 66). However, it should be noted that exercise may pose a risk to some older adults with chronic diseases. This study indicated that frail older adults had a higher risk of multiple chronic diseases than robust older adults. Furthermore, previous research suggested that older adults with multiple chronic diseases had an increased risk of depression (67).

We proposed that depression and poor sleep quality were potential early indicators of frailty. Building on the known findings that both sleep quality and depression were independently associated with frailty, we also considered the bidirectional association between depression and sleep quality. The existence of this bidirectional association may be explained by shared risk factors and pathophysiological mechanisms. Inflammation and hypothalamic–pituitary–adrenal axis (HPA) dysregulation have been proven as causative mechanisms of depression among older adults, and are closely associated with sleep disorders (16, 68, 69). Neuroendocrine dysregulation is associated with abnormal levels of insulin-like growth factor 1, testosterone, and cortisol, which is also a frequent underlying mechanism of frailty (70). Meanwhile, there is a positive association between frailty and levels of inflammatory cytokines, which are associated with depression and sleep quality, including interleukin 6 (IL-6) (71).

The mediation analysis revealed that depression had a mediating effect on the relationship between sleep quality and frailty, indicating that sleep quality impacted frailty not only directly, but also indirectly through depression. This finding was consistent with previous studies in Chinese community-dwelling older adults (27). Depression was found to play an important mediating role between sleep quality and frailty, with a mediation proportion of up to 60.4%. Sleep quality also mediated the association between depression and frailty in this study, with a mediation effect of 12.7%. Therefore, providing comprehensive care to prevent and improve frailty in older adults should not only focus on the physical aspects, but also on the psychological and lifestyle perspectives. Moreover, it is vital to take appropriate preventive measures for biological, psychological, and social factors that lead to frailty in order to enhance social functioning and life quality among the older adults (8). Healthy aging necessitates a collaborative effort from healthcare system, in addition to self-care of older adults and support from their families. We recommend early screening and intervention to identify potential frailty targets in order to promote healthy lifestyles and positive attitudes among older adults. This can help delay the onset of frailty or reverse its development in the older adults who have already experienced it. Ultimately, this research will lead to increased wellbeing among the older adults with frailty.

However, there are some limitations to this study. First, depression was assessed using a self-rating scale PHQ-9, and items of other scales were also mostly reported by the respondents. Although the use of self-report measure is common in epidemiological studies, it does increase the risk of reporting bias (72). Additionally, the screening scales we used for depressive symptoms and sleep disturbances cannot replace the diagnoses made by an experienced clinician. Diagnosing diseases requires a hospital visit or the utilization of specialized tools. Second, the PSQI is incapable of distinguishing between temporary and persistent sleep disturbances (73), necessitating the regular reassessment of sleep quality in older adults to grasp the changes and provide timely intervention measures. Third, frailty is a multidimensional indicator, including physical factors, psychological factors, and social factors (74). However, this study focused on physical frailty, lacking of the measurement of other dimensions. Fourth, it should be noted that causal associations cannot be inferred from the cross-sectional data used in this study (75). This study considered a bidirectional association between depression and sleep quality, but it cannot be ruled out that there may also be a bidirectional association between depression and frailty, as well as between sleep and frailty. The findings indicated that enhancing the psychological state and sleep quality of older adults might be a feasible strategy for improving frailty. Further studies are required to verify if there are any other associations between depression, sleep quality and frailty. In addition, the proportion of female participants in this study outweighed that of male ones, with females accounting for 65%. Nevertheless, the findings did not find any statistically significant differences in frailty among community older adults based on gender, and we also included it as a covariate for adjustment conducting mediation analysis. Last, this study mainly focused on the older adults who lived in the community, and, therefore, it would be inappropriate to extrapolate the findings to all older adults. In the actual process of the survey, some older adults with severe frailty might not be able to participate since it was not easy for them to get out of the house. Consequently, the obtained prevalence rate of frailty in the older adults may be lower than the actual prevalence rate.

## 5 Conclusion

In conclusion, the prevalence of frailty and pre frailty was as high as 35.1%. Age, consumptions of vegetables, exercise, sleep quality, depression, number of chronic diseases, chronic pain, and self-rated health were correlated with frailty. And this study revealed the bidirectional effect of depression and sleep quality, as well as the pathways contributing to frailty. Therefore, it is important to pay attention to frailty, depression, and sleep status of older adults. Timely intervention should be provided to those who suffered from frailty and pre frailty to improve their quality of life and reduce the burden on the medical system.

## Data availability statement

The raw data supporting the conclusions of this article will be made available by the authors, without undue reservation.

## Ethics statement

The studies involving humans were approved by ethics committee of School of Public Health, Fudan University (IRB00002408 and FWA00002399), and all participants or their respondents provided informed consent. The studies were conducted in accordance with the local legislation and institutional requirements. The participants provided their written informed consent to participate in this study.

## Author contributions

YZ: Conceptualization, Formal analysis, Methodology, Writing – original draft. GY: Formal analysis, Investigation, Writing – original draft. WB: Writing – review & editing. SW: Data curation, Investigation, Writing – original draft. XG: Data curation, Investigation, Writing – original draft. WZ: Data curation, Investigation, Writing – original draft. YL: Data curation, Investigation, Writing – original draft. YM: Data curation, Investigation, Writing – original draft. JG: Funding acquisition, Project administration, Writing – review & editing. WL: Funding acquisition, Project administration, Writing – review & editing. CK: Conceptualization, Methodology, Writing – review & editing.
