# Supplementary material for: Association of depression and sleep quality with frailty: a cross-sectional study in China
Source: Front Public Health. 2024 Apr 5;12:1361745. doi: 10.3389/fpubh.2024.1361745 (PMC11026860; doi:10.3389/fpubh.2024.1361745)
Supplement: Supplementary file 1 [file Data_Sheet_1.docx]

Supplementary materials

**Supplementary Table 1.** Direct, indirect, and total effects of associated variables on frailty.

| Path | Effect | *SE* | 95%CI | *p* |
| --- | --- | --- | --- | --- |
| Sleep quality⭢Depression⭢Frailty |  |  |  |  |
| Direct effect (c’) | 0.0216 | 0.0053 | 0.0112-0.0320 | <0.001 |
| Indirect effect (a$\times$b) | 0.0329 | 0.0043 | 0.0246-0.0416 | <0.001 |
| Total effect (c) | 0.0545 | 0.0049 | 0.0449-0.0641 | <0.001 |
| Depression⭢Sleep quality⭢Frailty |  |  |  |  |
| Direct effect (c’) | 0.0721 | 0.0055 | 0.0614-0.0829 | <0.001 |
| Indirect effect (a$\times$b) | 0.0105 | 0.0032 | 0.0044-0.0168 | <0.001 |
| Total effect (c) | 0.0826 | 0.0048 | 0.0731-0.0922 | <0.001 |

CI: Confidence intervals


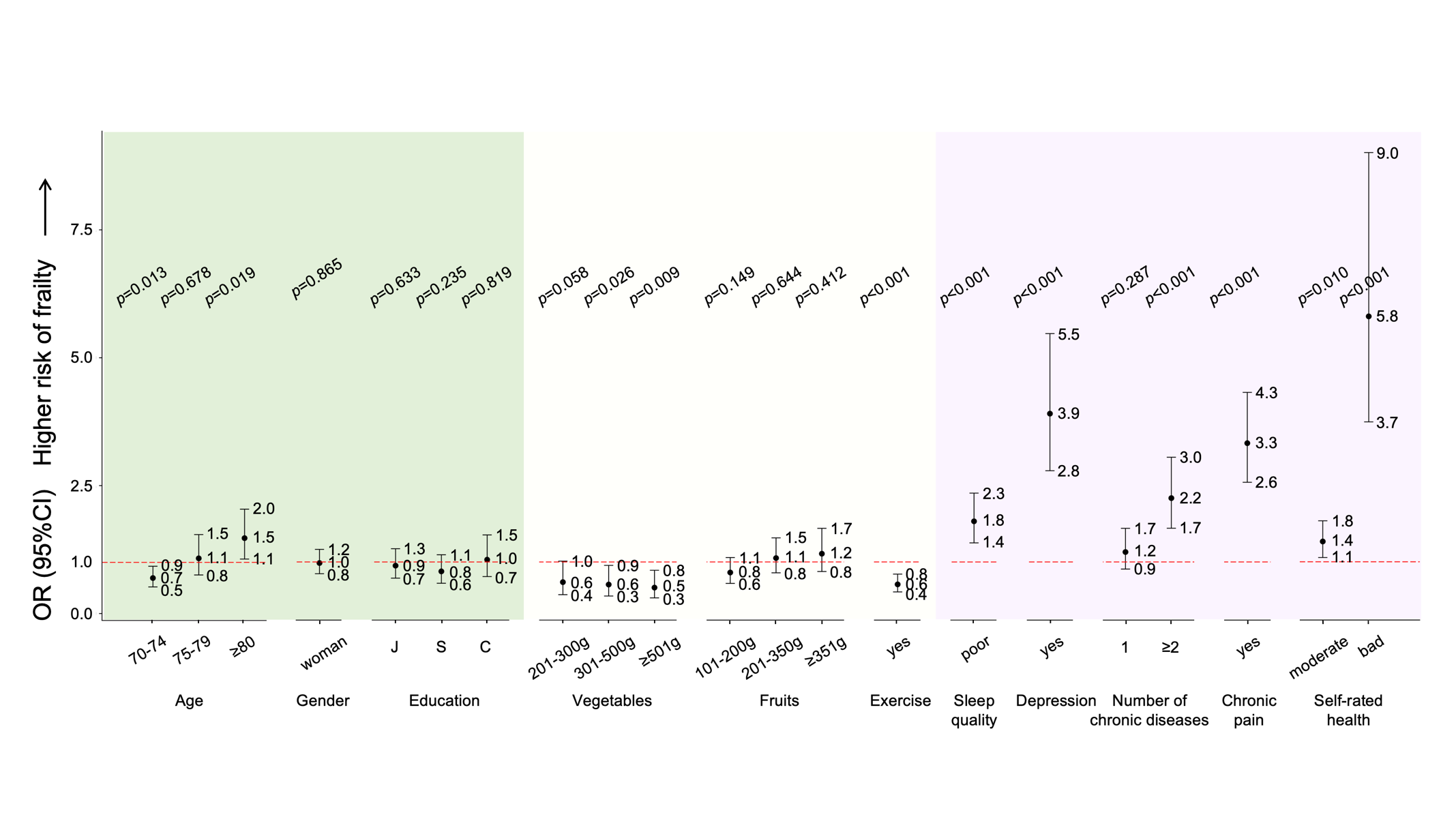


**Supplementary Figure 1.** OR for frailty in community-dwelling older adults by ordinal logistic regression analysis. OR: Odds ratio; g: gram; J: Junior middle school; S: Senior middle school/ technical secondary school; C: College degree and above
